# Training Global Oncologists: Addressing the Global Cancer Control Problem

**DOI:** 10.3389/fonc.2015.00080

**Published:** 2015-04-08

**Authors:** Surbhi Grover, Onyinye D. Balogun, Kosj Yamoah, Reinou Groen, Mira Shah, Danielle Rodin, Yehoda Martei, Adam C. Olson, Jeremy S. Slone, Lawrence N. Shulman, C. Norman Coleman, Stephen M. Hahn

**Affiliations:** ^1^Department of Radiation Oncology, University of Pennsylvania, Philadelphia, PA, USA; ^2^Department of Radiation Oncology, New York University Perlmutter Cancer Center, New York, NY, USA; ^3^Department of Radiation Oncology, Sidney Kimmel Cancer Center at Thomas Jefferson University, Philadelphia, PA, USA; ^4^Department of Gynecology and Obstetrics, Johns Hopkins Hospital, Baltimore, MD, USA; ^5^Department of Radiation Oncology, Henry Ford Health Systems, Detroit, MI, USA; ^6^Department of Radiation Oncology, University of Toronto, Toronto, ON, Canada; ^7^Department of Medicine, Division of Hematology/Oncology, Hospital of the University of Pennsylvania, Philadelphia, PA, USA; ^8^Department of Radiation Oncology, Duke University Medical Center, Durham, NC, USA; ^9^Baylor College of Medicine, Texas Children’s Hospital, Houston, TX, USA; ^10^Department of Medical Oncology, Dana-Farber Cancer Institute, Harvard Medical School, Boston, MA, USA; ^11^Radiation Research Program, Division of Cancer Treatment and Diagnosis, National Cancer Institute, Bethesda, MD, USA; ^12^International Cancer Expert Corps, Chevy Chase, MD, USA

**Keywords:** global health, oncology training, radiation oncology, surgical oncology, medical oncology

## Introduction

The global incidence of cancer has increased by approximately 20% in the past decade, an increase mostly due to cases in low- and middle-income countries (LMICs) (1). By 2020, up to 70% of the 20 million annual new cancer cases are expected to occur in LMICs (2). The incidence of cancer in LMICs is increasing rapidly; however, many countries are not prepared to address this epidemic. Cancer survival rates in LMICs are often less than one-third of those in high-income countries (3). In addition to local capacity-building efforts, the involvement of the oncology community from high-income countries will be instrumental in changing the course of this impending global cancer crisis.

There is a vital need to train global oncologists to work with colleagues in LMICs to develop sustainable capacity and infrastructure for clinical oncology care, research, and education. However, enumeration of specific goals and novel programs, and the path to implementing these programs, is not clear. Oncology programs in North America lack formal training or exposure to global oncology. Even without a formal curriculum, with the rise in global health (GH) oncology interest, several opportunities have developed for trainees committed to GH. We describe herein current opportunities and future directions for oncology trainees in the United States (US) who are interested in pursuing careers as global oncologists.

## Current Opportunities

### Medical and pediatric oncology

Many US training programs have responded to the growing interest in GH by offering international training opportunities. A survey of 380 US internal medicine residency programs showed that 57% of responding programs offered opportunities for international rotations (4). When selecting a residency program, 22% of pediatric residents indicated that GH training opportunities were essential/very important and 21% had a GH experience during pediatric residency (5). There has also been an increase in GH fellowships. A recent survey identified 80 GH fellowship programs, 14% of which were in internal medicine (6). For medical and pediatric oncology fellowships, there is scant information regarding trainee interest and the availability of international rotations. Few institutions have GH partnerships that offer opportunities for training, research, and career development in medical and pediatric oncology. The available opportunities are highlighted below.

1. The American Society of Clinical Oncology (ASCO) supports the Health Volunteers Overseas (HVO) program that recruits oncologists, faculty, and senior internal medicine residents to strengthen cancer care in LMICs. The program creates a forum for oncologists to share their medical expertise and build sustainable relationships with physicians addressing cancer care in these regions (7, 8).
2. Medicine residents pursue individually tailored curriculums in the form of short-term elective rotations at cancer centers in LMICs partnered with a US based institution, such as Botswana – University of Pennsylvania (9) and Yale/Stanford Johnson & Johnson Global Health Scholars Programs (10).
3. International pediatric oncology twinning programs through institutions like St. Jude Children’s Research Hospital, Children’s Hospital Boston/Dana-Farber Cancer Institute, and Texas Children’s Hospital/Baylor College of Medicine offer GH opportunities during pediatric hematology–oncology fellowship (11–13).
4. Graduates of internal medicine and pediatrics residencies can apply for a 1- to 2-year research and leadership role in clinical cancer care at the Butaro Cancer Center of Excellence (BCCOE) in Rwanda. They are mentored by local clinical leaders from Partners in Health, the Rwandan Ministry of Health and oncologists from the Dana-Farber Cancer Institute. More recently, funding has become available for a recent graduate of an oncology fellowship program to spend a year at the University Hospital at Mirebalais in Haiti (14).
5. The Academic Model for Providing Access to Healthcare (AMPATH) consortium, involving many North American centers led by Indiana University School of Medicine, offers oncology experience in Kenya through a partnership with Moi University School of Medicine and Moi Teaching and Referral Hospital (15).

Finally, for trainees and faculty members interested in global oncology in Africa, the African Organization for Research and Training in Cancer (AORTIC) conference brings together oncology professionals from Africa and abroad to present scientific work and participate in cancer education in Africa (16).

The existing requirements for medical and pediatric oncology fellowships in US by the Accreditation Council for Graduate Medical Education (ACGME) make active participation in global cancer activities during the core 3-year fellowship a challenge. Currently, significant barriers include the need for longitudinal patient care and on-site mentorship by board certified oncologists. For a fellow to spend significant time at a global site, it would likely need to be done after the core fellowship as an “unofficial” advanced fellowship, additional time that might thwart GH interest. Ideally, ACGME and individual programs will work together to incorporate time for optional GH activities into the core fellowship.

### Surgical oncology

Benefits of international rotations in LMICs for surgical residents include exposure to pathology distinct from that seen at the home institution, alternative disease management, cultural influences on disease presentation, and relationship building (17). For surgical oncology fellows specifically, the learning benefits of rotations in LMICs are even greater as cancer patients have fewer screening options, often present at advanced stages, might have different cancer biology, fewer imaging options, as well as limited perioperative care (18–20).

There are few residencies in general surgery and gynecology with an established global rotation. One such opportunity is the Paul Farmer Global Surgery fellowship, which is typically a 1- to 2-year fellowship following surgical residency, which gives recent graduates experience in GH research and surgical oncology in resource-poor settings, such as Haiti and Rwanda (21).

Of the 243 accredited U.S. obstetrics and gynecology residency programs, 34% offer formal didactics and 28% offer a formal rotation in global women’s health (22). Residents who participate in an international rotation are often very motivated to continue their involvement. However, once they start fellowship training opportunities to spend time abroad are limited. Of the 22 surgical oncology and 46 gynecologic oncology fellowship programs, only 1 program offers an international experience on their website. The Society of Gynecologic Oncology (SGO) welcomes international research and offers membership at reduced fees to international members from LMICs; a support program is also available for international attendees of the annual conference. However, no grants are available to our knowledge for fellows who are planning overseas research or work.

### Radiation oncology

While there is an increasing level of interest in GH oncology outreach among radiation oncology residents, available opportunities are very limited. A Global Health Interest Survey administered by the Association of Residents in Radiation Oncology (ARRO) in 2009 revealed that of 115 residents completing the survey, approximately 90% of respondents were interested in an international radiation oncology experience during their residency and 80% wished to incorporate international work in their future careers. However, <10% of the respondents had GH educational activities within their residency. Moreover, many residents noted barriers to an international rotation (funding, elective time, program director support) as well as unavailability of faculty guidance/mentorship at their home institution to foster GH interest (23).

The American Society of Radiation Oncology (ASTRO), in conjunction with ARRO and the American College of Radiology (ACR), promote international outreach by sponsoring travel grants. Annually, the ACR Foundation Goldberg-Reeder Resident Travel Grant awards $1500 to residents in order to spend at least 1 month assisting health care in a LMIC.

The ASTRO-ARRO Global Health Scholars Program also provides $2500 scholarships to selected residents for self-designed research, clinical or educational projects. Similarly, the Canadian Association of Radiation Oncology (CARO) has recently launched a GH scholarship for residents and fellows, which provides 2500 CAD for a clinical or research elective in a LMIC.

A few residency programs have incorporated international outreach into their curriculum. Through the Botswana-UPenn Partnership (24), residents at the University of Pennsylvania and elsewhere can provide clinical care in a LMIC. Similarly, the University of Chicago and the University of California-San Diego allocate time and financial resources for their senior residents to pursue rotations abroad.

At present, 84 LMICs have radiation therapy facilities. By 2020, over 12,000 radiation oncologists will be needed to staff radiation therapy facilities in these nations (25, 26). We posit that radiation oncology residents *can* play a role in addressing this disparity, particularly in this era of expanding remote telecommunication tools for quality assurance and treatment planning (27). Evidence suggests that short-term rotations are beneficial for both the resident and the international site providing opportunities for clinical care, knowledge exchange, research collaboration, and sustained partnerships. Through the ACR grant, residents have engaged in projects such as training staff to use brachytherapy units in Botswana (28, 29) and examining the survival rates of Ghanaian prostate cancer patients receiving external beam radiation therapy (30). We anticipate that longitudinal follow-up of the participants in the aforementioned programs and detailed reporting regarding their experiences will shed additional light on the specific benefits of these rotations.

## Going Beyond Training and a Career Path in Global Health

Interested trainees in GH provide a compassionate and forward thinking potential work force to address the disparity in oncology resources in LMICs. Yet, a well-defined career path is needed. Senior mentors and leaders worldwide are coming together to pilot a novel solution to GH in oncology. Signaling a transformational change that addresses both the global need for cancer care and the desire to create a sustainable altruistic component to physicians’ careers, the International Cancer Expert Corps (ICEC), recently established as an NGO, aims to reduce mortality and improve the quality of life for cancer patients in LMICs through structured support for dedicated faculty attempting to establish a formal career path, with metrics, for human service (31). This model will also cultivate faculty who will support residents pursuing their international health interests after training is completed.

## Conclusion

In recent years, a number of training programs have allowed oncology residents and fellows to participate in international electives and to enrich their GH perspective. These experiences have provided trainees exposure to clinical presentations of cancer and approaches to cancer treatment in diverse settings with varying availability of resources. However, a more systematic approach to mentorship in and learning about GH is needed, as well as a sustainable career path to effect change.

A proposed framework for a GH fellowship includes advanced course work such as a Masters of Public Health or degree in tropical medicine; domestic clinical medicine to maintain competencies and potential revenue creation; academic research; and international field work (32). Specifically for oncology, the curriculum should include (1) global risk factors and epidemiological trends across geographic regions and within tumor sites; (2) barriers to access to radiotherapy, chemotherapy, and surgery and innovative modalities that can be employed in different regions; (3) international clinical experience, with pre-departure training and post-visit debriefing; and (4) research training and experience in GH, supervised by local or international designated mentors with relevant expertise. These efforts can not only be directed toward education for both U.S. trainees and in-country professionals but also must be directed at implementation and implementation research. Training is a means to an end-provision of high-quality and affordable cancer care, and implementation of this care, is much more challenging than merely training. GH could be a specific module in all oncology training and possibly be integrated longitudinally within residency and fellowship programs. Such approaches would help to ensure that the GH perspective becomes a core competency of all graduating residents.

Global health training would benefit from a GH curriculum and a mechanism to connect trainees and faculty engaged in this field. GlobalRT is one such website (www.globalrt.org) that establishes an online global radiotherapy community (33). This site provides information about radiotherapy within the global context, ongoing projects, training experiences, and research within this area. The GlobalRT blog allows trainees and practitioners to post accounts of their experiences in any dimension of GH, allowing dialog and the exchange of information about opportunities in education, research, and clinical practice.

These initiatives are invaluable resources for oncology residents with an interest in GH. Their successes can inform the creation of a framework for training oncology residents to tackle GH issues. Furthermore, there is a need for the “clinical oncologist” skilled in multimodality cancer care who works closely with economists and experts in public health and policy to address the multi-factorial issues (34).

In conclusion, the global burden of cancer in LMICs is rising and it is imperative that the global oncology community contributes to a solution. Medicine is, by definition, a humanitarian endeavor, and bringing cancer care to those who have had no access should have an important place for all physicians including those practicing in resource-rich environments. There is increasing interest in global oncology among trainees. Although the opportunities are currently limited, trainees are demanding more exposure and to find ways to contribute to solving this global epidemic. We hope that increased focus on GH training in the future will better prepare trainees as global oncologists. To effectively address the global need and the rising trainee interest, fundamental changes to career paths are required, which would provide global benefit and serve as a model to address other non-communicable diseases.

## Conflict of Interest Statement

The authors declare that the research was conducted in the absence of any commercial or financial relationships that could be construed as a potential conflict of interest. TheAssociate Editor DanielGrant Petereit declares that, despite having collaborated with authors Surbhi Grover, Lawrence N. Shulman, C. Norman Coleman, and Stephen M. Hahn, the review process was handled objectively and no conflict of interest exists.
