# Corrigendum: “Training Global Oncologists: Addressing the Global Cancer Control Problem”

**DOI:** 10.3389/fonc.2015.00133

**Published:** 2015-06-10

**Authors:** Surbhi Grover, Onyinye D. Balogun, Kosj Yamoah, Reinou Groen, Mira Shah, Danielle Rodin, Yehoda Martei, Adam C. Olson, Jeremy S. Slone, Lawrence N. Shulman, C. Norman Coleman, Stephen M. Hahn

**Affiliations:** ^1^Department of Radiation Oncology, University of Pennsylvania, Philadelphia, PA, USA; ^2^Department of Radiation Oncology, New York University Perlmutter Cancer Center, New York, NY, USA; ^3^Department of Radiation Oncology, Sidney Kimmel Cancer Center at Thomas Jefferson University, Philadelphia, PA, USA; ^4^Department of Gynecology and Obstetrics, Johns Hopkins Hospital, Baltimore, MD, USA; ^5^Department of Radiation Oncology, Henry Ford Health Systems, Detroit, MI, USA; ^6^Department of Radiation Oncology, University of Toronto, Toronto, ON, Canada; ^7^Department of Medicine, Division of Hematology/Oncology, Hospital of the University of Pennsylvania, Philadelphia, PA, USA; ^8^Department of Radiation Oncology, Duke University Medical Center, Durham, NC, USA; ^9^Baylor College of Medicine, Texas Children’s Hospital, Houston, TX, USA; ^10^Department of Medical Oncology, Dana-Farber Cancer Institute, Harvard Medical School, Boston, MA, USA; ^11^Radiation Research Program, Division of Cancer Treatment and Diagnosis, National Cancer Institute, Bethesda, MD, USA; ^12^International Cancer Expert Corps, Chevy Chase, MD, USA

**Keywords:** global health, oncology training, radiation oncology, surgical oncology, medical oncology, corrigendum

Dr. Yehoda Martei was mistakenly left off the list of authors at the time of submission.

The author list should read as follows:

Surbhi Grover, Onyinye D. Balogun, Kosj Yamoah, Reinou Groen, Mira Shah, Danielle Rodin, Yehoda Martei, Adam C. Olson, Jeremy S. Slone, Lawrence N. Shulman, C. Norman Coleman, and Stephen M. Hahn.

The authors apologize for this omission in the original publication.

The original article has been updated.

## Conflict of Interest Statement

The authors declare that the research was conducted in the absence of any commercial or financial relationships that could be construed as a potential conflict of interest.

